# Supplementary material for: Predicting re-emergence times of dengue epidemics at low reproductive numbers: DENV1 in Rio de Janeiro, 1986–1990
Source: J R Soc Interface. 2020 Jun 24;17(167):20200273. doi: 10.1098/rsif.2020.0273 (PMC7328382; doi:10.1098/rsif.2020.0273)
Supplement: Supporting Information: Supporting Methods [file rsif20200273supp1.pdf]

Predicting re-emergence times of dengue epidemics at low reproductive numbers: DENV1 in Rio de Janeiro, 1986-1990.

Supporting Information: Supplemental Results and Materials and Methods

Rahul Subramanian, Victoria Romeo-Aznar,

Edward Ionides, Claudia T. Codeço, and Mercedes Pascual

June 1, 2020

Contents

1 Supplemental Methods: Analytical Skip Derivation **2**

1.1 Background 2

1.2 The dynamical system 3

1.3 Calculation of the critical value, S_C 4

2 Supplemental Methods: Stochastic Model Description **6**

2.1 Parameterization 6

2.1.1 Population Growth 7

2.2 Euler Simulation with Demographic Stochasticity 7

2.3 Environmental Stochasticity 9

2.4 Measurement Error 9

2.4.1 Notation and Observation Times for Observed Data in Stochastic Fitting 9
2.4.2 Monthly Accumulation of Expected Reported Cases . . . 10
2.4.3 Measurement Model 10
2.5 Model Fitting 11
2.6 Sensitivity Analysis 12

3 Supplemental Results: Sensitivity Analysis 12

4 Supplemental Results: Vector Model Considerations 14

5 Supplemental Figures 17

1 Supplemental Methods: Analytical Skip Derivation

1.1 Background

This skip analysis is based on [1]. The goal is, given the number of susceptible after an outbreak S_0, predict how long we should expect for a new epidemic, or equivalently, the number of skipping years that will occur. In Stone et al [1], a skipping event is when both the number of infectious and susceptible individuals increase in time. However, if the number of infectious individuals increases while the population of susceptible decreases, the event is defined as an outbreak.

A year is taken to have only two seasons and it starts in the Low season at times $t_{L\,n} = nT$ with $n \in \mathbb{N}_0$ and $T = 1\,year$. This is followed by the High season at times $t_{H\,n} = \frac{T}{2}(2n+1)$. Then, assuming $\beta = \beta_0(1 \pm \delta)$ (with $\beta = \beta_0(1-\delta)$ for the Low season and $\beta = \beta_0(1+\delta)$ for the High season), and no human population growth ($N = N_0 = const$, since the mortality and birth rates are equal, $\mu = \lambda$),

they obtain for the critical threshold for the susceptible population S_0

$$\frac{S_c(k)}{N} = s_c(k) = \frac{\gamma + \mu}{\beta_0} - \frac{(k+1)\mu}{2} \tag{1}$$

Finally, if $S_c(k+1) < S_0 < S_c(k)$, the disease will skip k years and an outbreak will emerge on the *k+1-th* year. For details on how they obtained Eq. (1) see the Supplementary Information of Stone et al [1].

1.2 The dynamical system

The SIR model

Equation (2) shows the SIR infectious model, where λ and μ are the birth and mortality rates, β the transmission rate and γ the recovery rate.

$$\begin{aligned} \tfrac{dS}{dt} &= \lambda N - \beta(t)\tfrac{SI}{N} - \mu S \\ \tfrac{dI}{dt} &= \beta(t)\tfrac{SI}{N} - \gamma I - \mu I \\ \tfrac{dR}{dt} &= \gamma I - \mu R \end{aligned} \tag{2}$$

From Eq. (1) the nullcline curve of S is defined as:

$$dS/dt = 0 = \beta S\left(\frac{\lambda N - \mu S}{\beta S} - \frac{I}{N}\right) \Rightarrow \frac{I*}{N} = \frac{\lambda - \mu S/N}{\beta S/N} \tag{3}$$

After an outbreak, $I << I*$ and the trajectory is below the S nullcline. Under this slow build dynamics of the system the susceptible differential equation on Eq. (2) may be approximated by

$$\frac{dS}{dt} = \beta\frac{SI*}{N} = \lambda N - \mu S \tag{4}$$

1.3 Calculation of the critical value, S_C

The assumptions

To obtain the critical value, $S_C(k)$ (Eq. (1)), Stone et al [1] consider no demographic growth, that is, $\lambda = \mu \implies N(t) = N_0 \,\forall t$. With this assumption the nullcline curve (Eq. (3)) is given by $i* = \mu(1-s)/(\beta s)$, where $i = I/N$ and $s = S/N$. Then they approximated $i*$ by

$$i* = \frac{\mu(1-s)}{\beta s} \simeq \frac{\mu}{\beta s} \implies \frac{ds}{dt} \backsimeq \mu \tag{5}$$

In this paper we proceed as in Stone et al. [1] but considering:

1. A sinusoidal transmission rate, $\beta = \beta_0(1 - \delta sin(\omega t))$ (see Fig. (17)).
2. Population demography, $N = N(t)$, $\lambda \neq \mu$.
3. And a $I*$ without approximation. The approximation on Eq. (5) is not always applicable and in particular gives some contradictory results when $r_0 = \beta_0/(\gamma + \mu) < 1$. Specifically the threshold s_c shown on Eq.(1) allows outbreaks even when $r_0 < 1$.

The calculation

When $I << I*$, that is, in the period of the slow build dynamics, the set of differential equations of the system is

$$\begin{aligned} \tfrac{dS}{dt} &= \lambda N - \mu S \\ \tfrac{dI}{dt} &= \beta(t)\tfrac{SI}{N} - \gamma I - \mu I \\ \tfrac{dN}{dt} &= (\lambda - \mu)N \end{aligned} \tag{6}$$

where, because $N = S + I + R$, we have replaced the equation of the number of individuals recovered by the equation of the total population. By defining,

$Z = \frac{\beta_0}{\gamma+\mu} S = r_0 S$ and $X = \ln(I)/(\gamma+\mu)$, and solving the equation of $\dot{N}$ with $N_0 = N(t = t_0)$, we obtain the following system, equivalent to Eq. (6)

$$\begin{aligned} \dot{Z} + \mu Z &= r_0 \lambda N = r_0 \lambda N_0 e^{(\lambda-\mu)(t-t_0)} \\ \dot{X} &= (1 - \delta \sin(\omega t)) \tfrac{Z}{N_0} e^{-(\lambda-\mu)(t-t_0)} - 1 \end{aligned} \tag{7}$$

The variable Z in Eq. (7) is described by a first order differential equation which can be solved with standard techniques, and Eq. (8) shows the result

$$Z(t) = (Z_0 - r_0 N_0) e^{-\mu(t-t_0)} + r_0 N(t) \tag{8}$$

where $Z_0 = Z(t = t_0)$. By replacing Eq. (8) on Eq. (7), defining $z_0 = Z_0/N_0$, and taking $t_0 = 0$, we obtain

$$\dot{X} = (r_0 - z_0) e^{-\lambda t} \delta \sin(\omega t) - \delta r_0 \sin(\omega t) - (r_0 - z_0) e^{-\lambda t} + r_0 - 1 \tag{9}$$

Then, the strategy is to evaluate the sign of the integral between t_0 (which has been taken as 0 without lost of generality) and $t_{H\,n} = \frac{T}{2}(2n+1)$ (the high season starting time) of Eq. (9). In particular, by zeroing this integral, the following expression for S_C is obtained,

$$s_C = \frac{S_c}{N_0} = 1 + \frac{\pi(2n+1)(1 - 1/r_0) - 2\delta}{\omega f(\omega, \delta, \lambda, n)} \tag{10}$$

where $f(\omega, \delta, \lambda, n) = \left(1 + e^{-\lambda t_{H\,n}}\right) \frac{\omega\delta}{\omega^2+\lambda^2} - \left(1 - e^{-\lambda t_{H\,n}}\right) \frac{1}{\lambda}$.

2 Supplemental Methods: Stochastic Model Description

2.1 Parameterization

The stochastic model is an approximation of the deterministic model used for the skip analysis. For simplicity and given the short time interval, we assume that there is no population growth over the two and half years of the DENV1 invasion ($r = \mu_{\mathrm{H}}$) and that births and deaths occur at rate $\mu_{\mathrm{H}} = \frac{1}{74.49*365}\mathrm{day}^{-1}$ (the inverse of the average life expectancy in Brazil according to the 2010 census [2] expressed in units of days). Note that population growth is re-introduced in numerical simulations below that consider a longer time period. We assume that there are no recovered individuals at the start of the epidemic, so all other individuals in the population not initially infected are susceptible. The recovery rate γ is fixed at $\frac{1}{17}\mathrm{day}^{-1}$, assuming an exponentially distributed duration of infection with mean 17 days. We then profile over the recovery rate to verify that this fixed value of γ is plausible (see Sensitivity Analysis). We set $\omega = \frac{2\pi}{365}\mathrm{day}^{-1}$. The numerical solution to the stochastic model is a discrete-time model with fixed time step $\Delta t = 1$ day, and a discrete state space (i.e. the number of people in each compartment S, I, and R at any point in time must be integers). During simulation, the number of individuals who will move from one compartment to another over the course of each day is calculated via Euler simulation.

We first describe the Euler approximation for an ordinary differential equation. Then, we explain how we add demographic stochasticity (the consequence of a process acting probabilistically on a collection of discrete individuals) and environmental stochasticity (fluctuations in process rates depending on random, or unmodeled, effects that act at a population level). Finally, we specify how we model measurement noise (error in measuring the true number of dengue cases

each month taking into account both a fixed mean rate of under-reporting and additional variation).

2.1.1 Population Growth

Recall that the term r represents the per capita rate at which new individuals enter the population, while $\mu_{\mathrm{H}}$is the death rate. Let h represent the per capita growth rate of the population (i.e. $h = r - \mu_{\mathrm{H}}$). We assume that the net population growth rate is exponential:

$$\frac{dN}{dt} = hN \tag{11}$$

Census population estimates from 1991[3] and 2000[4] were used to calculate h assuming exponential growth. This rate was used to interpolate the population in 1986. In the implementation of the stochastic model, the population growth over each time step was fed in as a covariate using the R package pomp.

2.2 Euler Simulation with Demographic Stochasticity

We simulate the underlying SIR process model with fixed time steps of constant size Δt to obtain estimates of compartment sizes at time t ($\tilde{S}(t)$, $\tilde{I}(t)$, and $\tilde{R}(t)$). The model incorporates demographic stochasticity into the Euler approximations to obtain integer state variable values after each time step. For example, suppose that we want to calculate the number of individuals who move from the susceptible to the infected class over the course of a time step. Let $\mu_{\mathrm{SI}}(t)$ represent the instantaneous rate at which individuals move from the susceptible to the infected compartment. Under the deterministic model, we have:

$$\mu_{\mathrm{SI}}(t) = \beta(t)(\frac{I(t)}{N}) \tag{12}$$

Let $\Delta\tilde{N}_{\mathrm{SI}}$ represent the Euler approximation for the number of individuals who move from the susceptible to infected compartment between time step t and time step $t+\Delta t$. We assume that $\Delta\tilde{N}_{\mathrm{SI}}$ is drawn from a binomial distribution with exponentially decaying probability:

$$\Delta\tilde{N}_{\mathrm{SI}} \sim Binomial(\tilde{S}(t), 1 - e^{-\mu_{\mathrm{SI}}(t)\Delta t}) \tag{13}$$

All of the instantaneous deterministic transition rates are shown below. The symbol $\bullet$ denotes transitions entering and leaving the study population (i.e. $\bullet S$ denotes births and $S\bullet$ deaths):

$$\mu_{\mathrm{SI}}(t) = \beta(t)(\frac{\tilde{I}(t)}{N}) \tag{14}$$

$$\mu_{\mathrm{IR}}(t) = \gamma \tag{15}$$

$$\mu_{\bullet\mathrm{S}}(t) = r \tag{16}$$

$$\mu_{\mathrm{S}\bullet}(t) = \mu_{\mathrm{I}\bullet}(t) = \mu_{\mathrm{R}\bullet}(t) = \mu_{\mathrm{H}} \tag{17}$$

Euler approximations for all compartment transitions are shown below:

$$\Delta\tilde{N}_{\mathrm{SI}} \sim Binomial(\tilde{S}(t), 1 - e^{-\mu_{\mathrm{SI}}(t)\Delta t}) \tag{18}$$

$$\Delta\tilde{N}_{\mathrm{IR}} \sim Binomial(\tilde{I}(t), 1 - e^{-\mu_{\mathrm{IR}}(t)\Delta t}) \tag{19}$$

$$\Delta\tilde{N}_{\bullet\mathrm{S}} \sim Binomial(\tilde{N}(t), 1 - e^{-\mu_{\bullet\mathrm{S}}(t)\Delta t}) \tag{20}$$

$$\Delta\tilde{N}_{\mathrm{S}\bullet} \sim Binomial(\tilde{S}(t), 1 - e^{-\mu_{\mathrm{S}\bullet}(t)\Delta t}) \tag{21}$$

$$\Delta\tilde{N}_{\mathrm{I}\bullet} \sim Binomial(\tilde{I}(t), 1 - e^{-\mu_{\mathrm{I}\bullet}(t)\Delta t}) \tag{22}$$

$$\Delta\tilde{N}_{\mathrm{R}\bullet} \sim Binomial(\tilde{R}(t), 1 - e^{-\mu_{\mathrm{R}\bullet}(t)\Delta t}) \tag{23}$$

Using these expressions, we can write an expression for the number of people in each compartment at the end of each time step (time $t + \Delta t$)

$$\tilde{S}(t + \Delta t) = \tilde{S}(t) - \Delta \tilde{N}_{\mathrm{SI}} + \Delta \tilde{N}_{\bullet \mathrm{S}} - \Delta \tilde{N}_{\mathrm{S}\bullet} \tag{24}$$

$$\tilde{I}(t + \Delta t) = \tilde{I}(t) + \Delta \tilde{N}_{\mathrm{SI}} - \Delta \tilde{N}_{\mathrm{IR}} - \Delta \tilde{N}_{\mathrm{I}\bullet} \tag{25}$$

$$\tilde{R}(t + \Delta t) = \tilde{R}(t) + \Delta \tilde{N}_{\mathrm{IR}} - \Delta \tilde{N}_{\mathrm{R}\bullet} \tag{26}$$

2.3 Environmental Stochasticity

As stated in the Materials and Methods section, environmental noise (variation in the transmission rate $\beta(t)$ due to random environmental variation) was captured via multiplicative gamma white noise in the transmission rate as described by [5]. On time step size Δt we multiplied the transmission rate by $\frac{\Delta\Gamma}{\Delta t}$ where $\frac{\Delta\Gamma}{\Delta t}$ was drawn from a Gamma distribution with mean 1 and variance $\frac{{\sigma_P}^2}{\Delta t}$.

The gamma white noise method used in this model is further described in [5].

2.4 Measurement Error

2.4.1 Notation and Observation Times for Observed Data in Stochastic Fitting

The observed data $Y_{1:\mathrm{M}}$ represent monthly observed case counts in the city of Rio de Janeiro taken at times $t_{\mathrm{OBS}_1}, t_{\mathrm{OBS}_2} ... t_{\mathrm{OBS}_M}$, where $M = 26$ observations. Observation Y_m in that sequence was taken at time t_{OBS_m} and is the number of observed infections reported between time t_{OBS_m} and time $t_{\mathrm{OBS}_{m-1}}$. Time t_{OBS_0} corresponds to the start time for the simulation of the initial epidemic (May 1, 1986). In the stochastic model simulation, the time variable t is defined as the number of days since January 1, 1986. Therefore, $t_{\mathrm{OBS}_0} = 120$. The

first observation used in the fitting is then observation Y_1 measured at time $t_{\mathrm{OBS}_1} = 151$ (June 1, 1986). Note that in the raw data, the date for Y_1 is listed as "May 1986", corresponding to the total number of cases observed by the local health system between May 1, 1986 and May 30, 1986. The last observation used is $t_{\mathrm{OBS}_M} = t_{\mathrm{OBS}_{26}} = 912$ (July 1, 1988).

2.4.2 Monthly Accumulation of Expected Reported Cases

Let C_m represent the true number of reported cumulative cases for each month at observation time t_{OBS_m}.

Recall that during the Euler Simulation of the stochastic model, the number of individuals who move from from Susceptible S to Infected I is calculated for every time step of size Δt. This is the quantity $\Delta\tilde{N}_{\mathrm{SI}}$ from Section 2.3.

For ease of notation, let us use U_t to denote the value of $\Delta\tilde{N}_{\mathrm{SI}}$ calculated for the time step between time t and time $t + \Delta t$.

We can write an expression for C_m as the summation of the $\Delta\tilde{N}_{\mathrm{SI}}$ values over all of those time steps in month m. Formally:

$$C_m = \sum_{t=t_{\mathrm{OBS}_{m-1}}}^{t_{\mathrm{OBS}_m}} U_t \tag{27}$$

In the pomp implementation of the model, this issue is dealt with by designating the variable C as an accumulator variable via the accumvars() argument to the pomp constructor. The true number of reported cases are then accumulated for each (monthly) observation.

2.4.3 Measurement Model

The measurement model relates how the true number of cumulative cases for each month C_m relates to the observed number of monthly cases Y_m.

The formulation of the model takes into account under-reporting via the reporting rate parameter ρ. However, the true reporting rate may not necessarily be constant in time, for example, as the epidemic spreads to new neighborhoods within the city of Rio or as the surveillance system is constructed and activated. To take into account additional over-dispersion in the observed case data, we incorporate a measurement model in which we assume that the observed number of monthly dengue cases (Y_m) (the observed data) at time t corresponding to the end of a reporting month are drawn from a negative binomial distribution with mean equal to the true number of cases multiplied by the reporting rate ρC_m and dispersion parameter σ_{M}. The mean of the distribution is $\mu = \rho C_m$ while the variance can be written as $\mu + \sigma_{\mathrm{M}}^2 \mu^2$. Thus, when the dispersion parameter is close to zero ($\sigma_{\mathrm{M}} \to 0$), the measurement model reduces to a Poisson distribution.

2.5 Model Fitting

The model was fit using the mif2 method in the R-package pomp. The mif2 method implements the iterated filtering algorithm known as IF2 and described in (50). For each parameter being profiled, the profileDesign function in the pomp package was used to generate a set of starting points at 30 different evenly spaced values within prescribed ranges. The function created 40 different initial sampling points drawing from a box given by the boundaries of the original parameter range. For example, for the I_0 profile, a set of 30 starting points evenly spaced between 1 and 10,000 was generated. For each of those 30 starting points, the profileDesign function created 40 different initial sampling points with the same value of I_0 but different values for the other parameters being fitted (β_0, δ, γ, ϕ, σ_{M}, and σ_{P}) where the different values were uniformly drawn from the boundaries for those parameters in the original box. This yielded a

total of 1200 starting points for each parameter profile. The mif2 search from each starting point was repeated five independent times.

2.6 Sensitivity Analysis

In the sensitivity analysis, we consider two alternate models to the SIR Cosine model: the SIR Spline Model, and the SEIR Spline Model. The SIR Spline Model was an SIR model like the main stochastic model but assumed that the transmission rate was a function of three periodic cubic splines instead of a cosine function (SIR Spline Model). The spline coefficients b_1, b_2, and b_3 were fit instead of the cosine-function parameters. The second alternate model used splines but had an additional exposed class (the SEIR Spline Model) and an additional fitted initial value parameter (E_0). The duration of the incubation period was fixed at 10 days and the duration of infection at 7 days so that both periods sum to the value of the infection period fixed for the SIR Spline and Cosine Models. During the fitting of all four models, the duration of infection was fixed (at 17 days in the SIR Models and 7 days in the SEIR Models). The second part of the sensitivity analysis was a profile of the recovery rate using the SIR Cosine Model. We used a range of recovery rates corresponding to durations of infection between 2-22 days.

3 Supplemental Results: Sensitivity Analysis

Adding an incubation period or altering the form of seasonality in the model did not alter parameter estimates for R_0, reporting rate ρ and amplitude of seasonal transmission δ better explain observed dynamics. All three models (SIR with Cosine Seasonality, SIR with Spline Seasonality, and SEIR with Spline Seasonality) gave similar parameter estimates and likelihoods with respect to 2.5 years of observed cases from 1986-1988 (Supplemental Table ST3). The SEIR model

had a higher AIC score but a narrower profile for the environmental process noise standard deviation σ_{P} (Supplemental Figure 5, Supplemental Tables ST2 and ST3) compared to the flat σ_{P} profiles of other models. Selected profiles for all models are included in Supplemental Figures 4, 5, 6, and 7. An additional profile for the transmission phase parameter ϕ for the SIR Cosine Model is included in Supplemental Figure 8.

The flatness of the environmental process noise standard deviation (σ_{P} profile and high σ_{P} values for all parameter combinations with non-zero re-emergence probabilities (Figure 5) may both be additional indicators of model misspecification. Analysis of filter trajectories showed that the maximum likelihood estimate parameter combination in the SIR Cosine Model, which did not have high σ_{P} could not easily explain the rapid decrease in dengue transmission in beginning of the transmission season in 1988 (Supplemental Figure 12). Some parameter combinations with high σ_{P}outperformed the maximum likelihood estimate in capturing this decrease (Supplemental Figure 12), but under-performed the maximum likelihood estimate in capturing the peak during the second year of the epidemic (Supplemental Figure13). More generally, the increased variance of the large process noise parameter combination may impact predictive utility. Varying the duration of infection did not substantially reduce the expected time to re-emergence. During the principal analysis fitting, the duration of infection was fixed at 17 days in the SIR Models and 7 days in the SEIR Models. As an additional sensitivity analysis, we profiled over the recovery rate γ for the SIR Cosine Model (Supplemental Figure 9) with a range of recovery rates corresponding to durations of infection of between 2-22 days. Based on the range of parameter combinations in the profile within 2 log likelihood units of the profile peak, durations of infection between 5 and 20 days for the SIR Cosine model were supported by the data. The expected number of skips was re-calculated

using all parameter combinations within 2 log likelihood units of the γ profile maximum and ranged from 25 to over 100 years (Supplemental Figure 10). Parameter combinations with re-emergence times longer than 100 years had values of R0<1 (Supplemental Figure 10), high reporting rates (up to 30%), and substantial process noise (Supplemental Figures 10 and 11). The support for these deterministically implausible parameterizations may be an artifact of the short length of the time series and the large magnitude of the process noise.

4 Supplemental Results: Vector Model Considerations

Epidemic model

The mosquito-human coupled dengue model considered follows the equations of [6] but without the 'exposed' class compartment. Our purpose is to illustrate that explicit consideration of the vector is consistent with the seasonal transmission rate we have adopted in our SIR model, in particular in terms of its basic shape. For this purpose, we rely on a simple representation of the mosquito component, with two classes as in many modeling studies ([7, 8, 9, 10, 11]) . Given the temperature variation in Rio de Janeiro, the duration of the omitted exposed class would be short, and the typical mosquito lifespan would not preclude transmission.

In the model, the mosquitoes M can be susceptible W or infectious Z:

$$\frac{dW}{dt} = g(T)\, M(1 - \frac{M}{K}) - a(T)\, pMI(T)\, W \frac{I}{N} - \mu_M W \tag{28}$$

$$\frac{dZ}{dt} = a(T)\, pMI(T)\, W \frac{I}{N} - \mu_M Z \tag{29}$$

where a is the biting rate, μ_M is the adult mortality rate and pMI is the prob-

ability of transmission. The function $g(T) = EFD(T)\, pEA(T)\, MDR(T)\, \mu_M^{-1}$ depends on mosquitoes parameters: EFD corresponds to the eggs laid per female per gonotrophic cycle (number/female), pEA represents the mosquito egg-to-adult survival probability, and MDR is the mosquito egg-to-adult development rate (1/days). The human population is described by the following equation:

$$\frac{dS}{dt} = -a(T)\, b(T)\, Z \frac{S}{N} \frac{dI}{dt} = a(T)\, b(T)\, Z \frac{S}{N} - \gamma I \frac{dR}{dt} = \gamma I \tag{30}$$

We considered a mosquito mortality rate independent from temperature. This choice follows from the known difficulty in mapping laboratory thermal curves for this parameter to those from the field [9] . In particular, field observations exhibit constancy at intermediate values of temperature (including the range of variation in Rio de Janeiro) rather than the typical bell-shaped curves from the laboratory.

By considering a quasi-static approximation to Eq. (29) and $\hat{W} \simeq \hat{M}$ [12], we obtain the following expression for $\hat{Z}$,

$$\hat{Z} \simeq \frac{a(T)\, pMI(T)}{\mu_M} \frac{\hat{M}\, I}{N} = \frac{a(T)\, pMI(T)}{\mu_M} \frac{I}{N} K(1 - \frac{\mu_M}{g(T)}) \tag{31}$$

, where $\hat{M} = K(1 - \frac{\mu_M}{g(T)})$ is obtained by zeroing the sum of Eq.(28) and Eq.(29) since $M = W + Z$. Then, we incorporate Eq.(31) in Eq.(30) and we obtain the following effective transmission rate β_{eff}

$$\beta_{eff} \simeq a(T)^2\, b(T)\, pMI(T)\, \mu_M^{-1} \frac{K}{N}(1 - \frac{\mu_M}{g(T)}) \tag{32}$$

Comparison of the transmission rates

To compare the behavior of the expression shown in Eq. (32) with the transmission rate obtained from the fitted SIR cosine model, we use a cosine function to describe the temperature of Rio de Janeiro. The dots in Fig. 19A show the weekly temperature reported in [13] and the solid line correspond to $TempSim(t) = 25 + 5\,cos(2\pi t/365 - 0.5)$.

The transmission rate obtained from the cosine temperature and the Eq. (32) (values of the parameters are taken from [14] and [15]) is shown on Fig. 19B, as well as the transmission rate of the fitted SIR cosine model. The values have been rescaled between 0 and 1 for a better comparison of the curves.

5 Supplemental Figures

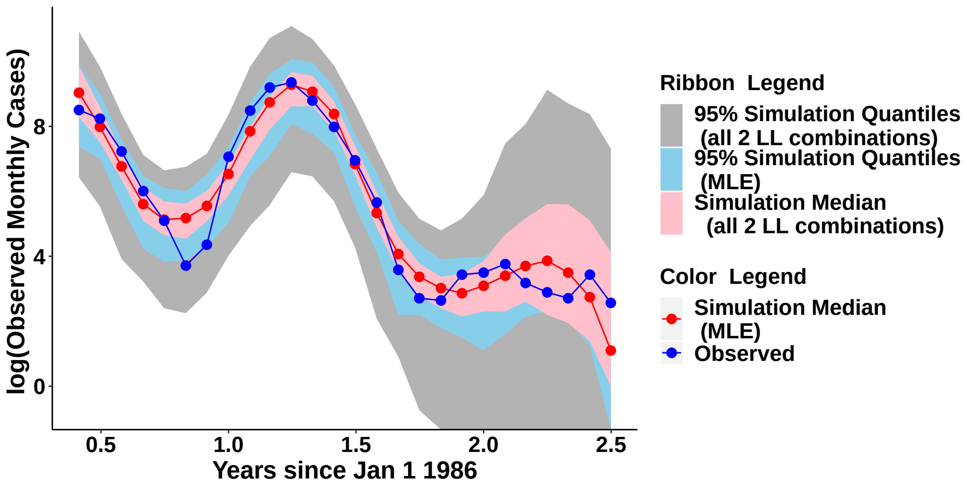

Figure 1: **Observed vs simulated cases from parameter combination for the stochastic SIR Cosine Model, fit to 2.5 years of DENV1 case data (fixed recovery rate)** Log of observed monthly cases from April 1986 to June 1988 are shown in blue. Simulated cases were estimated from 100 simulations for each parameter combination within 2 log-likelihood units of the highest likelihood parameter combination (the MLE). Median values from 100 simulations from the MLE the are shown in red. The range of simulation medians across all parameter combinations within 2 log-likelihood units is shaded pink. The shaded blue region denotes the 95% quantile boundaries across all 100 simulations from the MLE. The shaded grey region denotes 95% quantile boundaries from all simulations (across all parameter combinations within 2 log-likelihood units of the MLE).

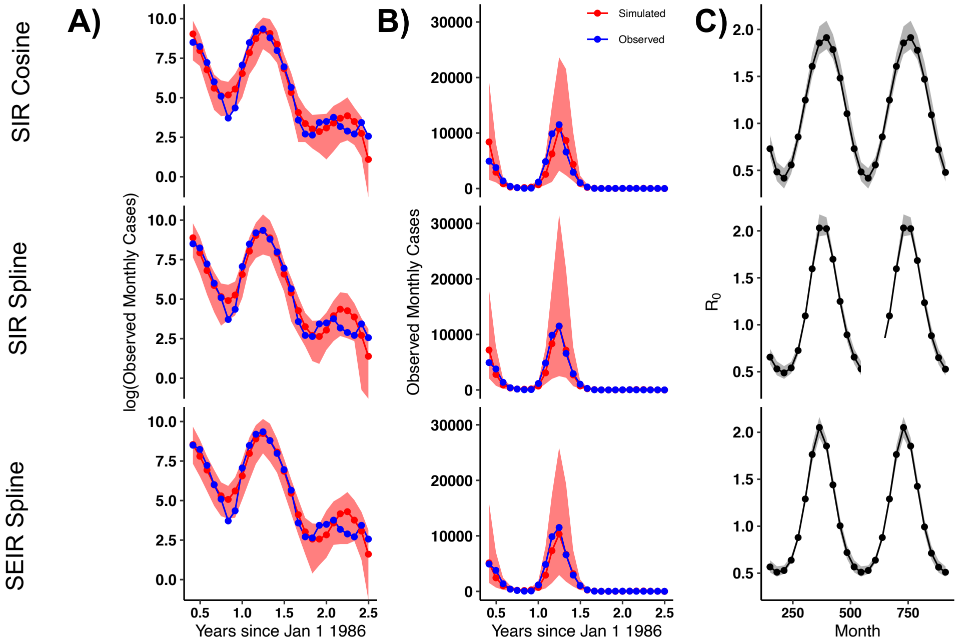

Figure 2: **A-B Comparison of simulated values with the fitted model and observed data on a log (A) and regular (B) scale.)** Observed monthly cases from April 1986 to June 1988 are shown in blue. Median values from 100 simulations with the maximum likelihood parameter combination are shown in red. The shaded red region denotes the 2.5% and 97.5%th quantile boundaries from those simulations. **C) Estimates for R0(t).** The black line denotes the trajectory of R0(t) for the maximum likelihood estimate. The shaded grey region represents the 2.5% and 97.5%th quantile boundaries for trajectories from all parameter combinations within 2 log likelihood units of the maximum likelihood estimate. Each parameter combination has only one seasonal trajectory for R0(t) since R0(t) is a deterministic quantity. Results for three models are shown: the SIR Cosine Model (described in the main manuscript) as well as an SIR Spline and SEIR Spline Model.

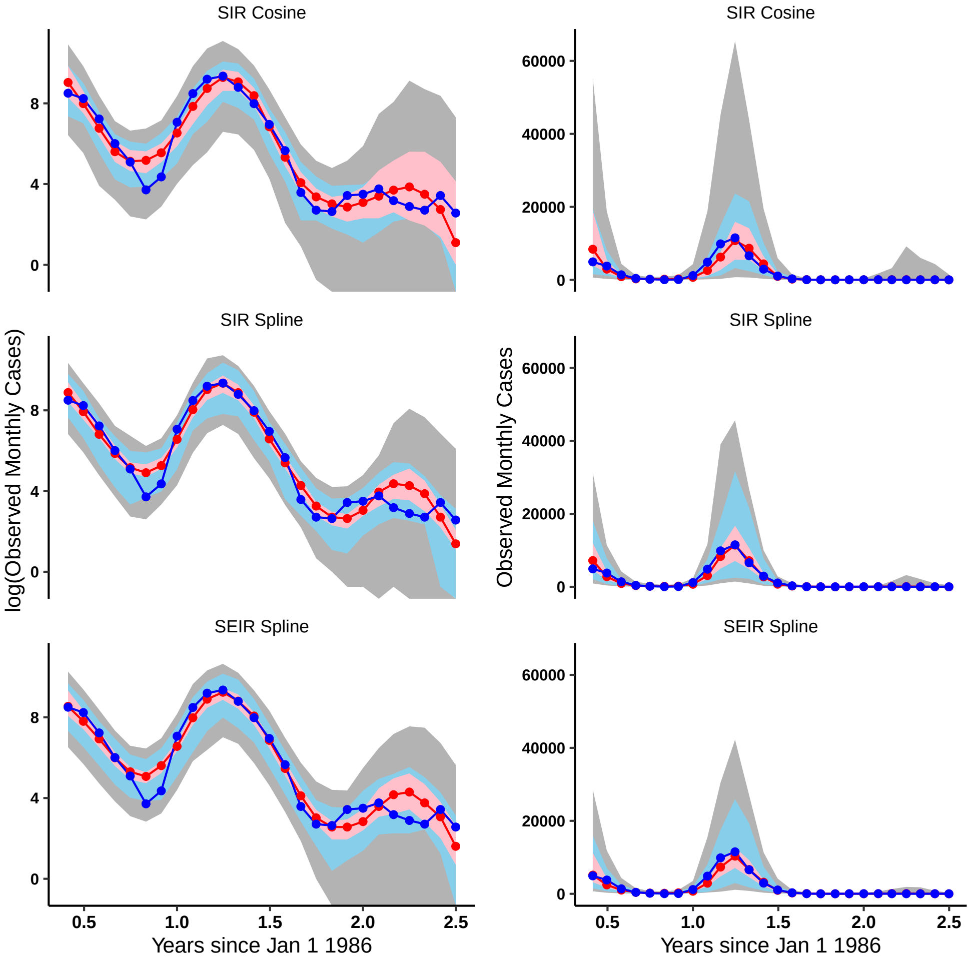

Figure 3: **Comparison of all examined parameter combinations within 2 log likelihood units of the maximum likelihood estimate for each fitted model with observed dengue case counts for each of 3 models.** For each parameter combination, 100 independent stochastic simulations were conducted. The right hand panel shows cases on a standard scale, while the left hand panel is on a log scale. Log of observed monthly cases from April 1986 to June 1988 are shown in blue. Simulated cases were estimated from 100 simulations for each parameter combination within 2 log-likelihood units of the highest likelihood parameter combination (the MLE). Median values from 100 simulations from the MLE the are shown in red. The range of simulation medians across all parameter combinations within 2 log-likelihood units is shaded pink. The shaded blue region denotes the 95% quantile boundaries across all 100 simulations from the MLE. The shaded grey region denotes 95% quantile boundaries from all simulations (across all parameter combinations within 2 log-likelihood units of the MLE).

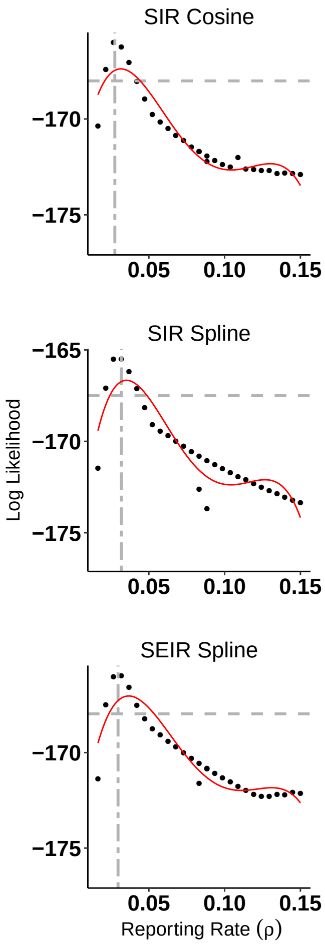

Figure 4: **Profiles of reporting rate (ρ) for all three models.** The red curve is a polynomial fit to the subset of the profile points shown on the figure. The single dashed grey horizontal line represents the likelihood value 2 log likelihood units below the peak of the profile. This line provides an estimate of confidence intervals for the given parameter. The grey vertical line denotes the parameter value of the maximum likelihood estimate across all parameter profiles for that model.

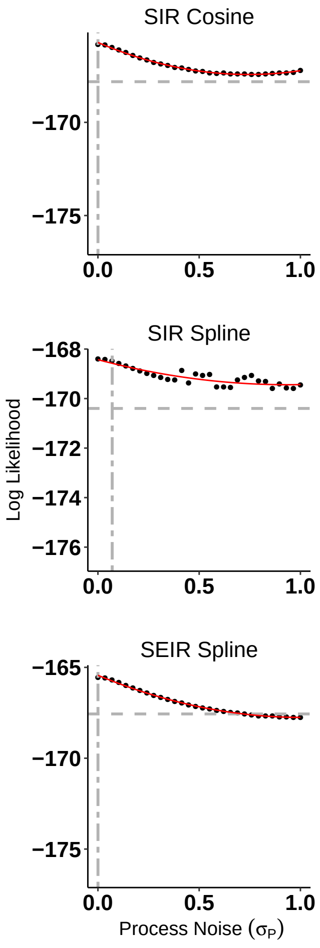

Figure 5: **Profiles of the environmental process noise magnitude parameter ($\sigma_{\mathbf{P}}$) for all three models.** The red curve is a polynomial fit to the subset of the profile points shown on the figure. The single dashed grey horizontal line represents the likelihood value 2 log likelihood units below the peak of the profile. This line provides an estimate of confidence intervals for the given parameter. The grey vertical line denotes the parameter value of the maximum likelihood estimate across all parameter profiles for that model.

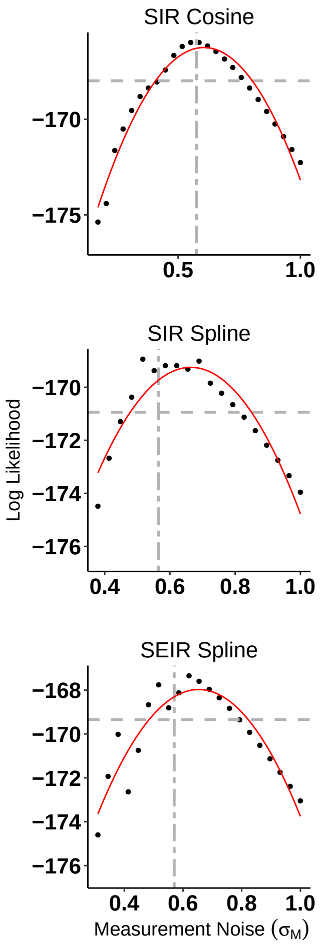

Figure 6: **Profiles of the measurement noise magnitude parameter (σ_{M}) for all three models.** The red curve is a polynomial fit to the subset of the profile points shown on the figure. The single dashed grey horizontal line represents the likelihood value 2 log likelihood units below the peak of the profile. This line provides an estimate of confidence intervals for the given parameter. The grey vertical line denotes the parameter value of the maximum likelihood estimate across all parameter profiles for that model.

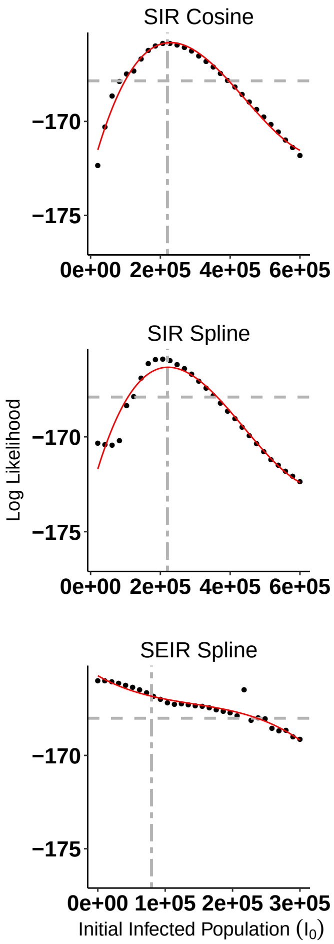

Figure 7: **Profiles of the initial number of infected people at the start of the simulation (I_0) for all three models.** The red curve is a polynomial fit to the subset of the profile points shown on the figure. The single dashed grey horizontal line represents the likelihood value 2 log likelihood units below the peak of the profile. This line provides an estimate of confidence intervals for the given parameter. The grey vertical line denotes the parameter value of the maximum likelihood estimate across all parameter profiles for that model.

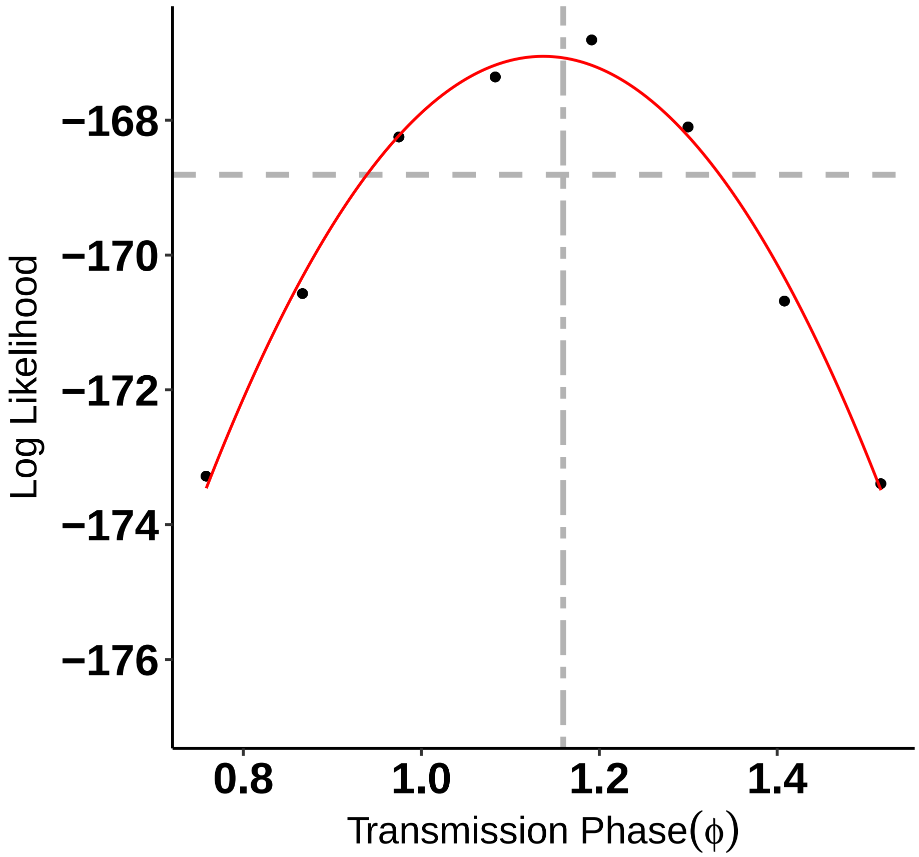

Figure 8: **Profiles of the phase parameter (ϕ) for the SIR Cosine Model.** The red curve is a polynomial fit to the subset of the profile points shown on the figure. The single dashed grey horizontal line represents the likelihood value 2 log likelihood units below the peak of the profile. This line provides an estimate of confidence intervals for the given parameter. The grey vertical line denotes the parameter value of the maximum likelihood estimate across all parameter profiles for that model.

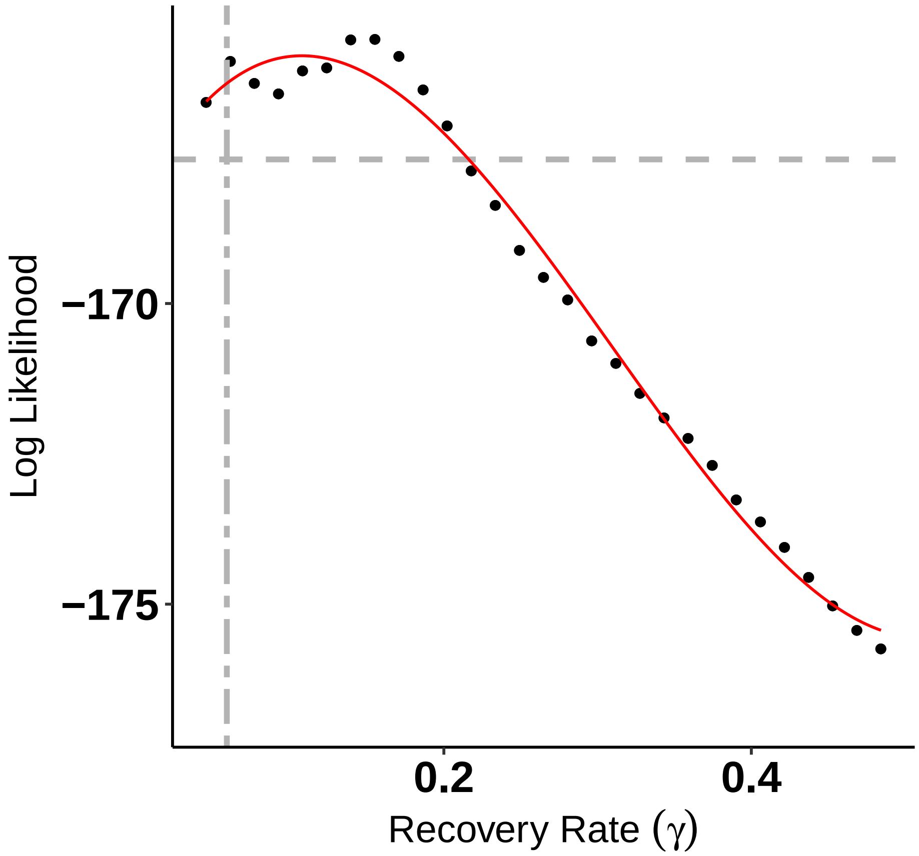

Figure 9: **Profiles of the recovery rate (γ) for the SIR Cosine Model.** The red curve is a polynomial fit to the subset of the profile points shown on the figure. The single dashed grey horizontal line represents the likelihood value 2 log likelihood units below the peak of the profile. This line provides an estimate of confidence intervals for the given parameter. The grey vertical line denotes the value of gamma that was used for the original fit of the SIR Cosine Model (where the recovery rate was fixed) .

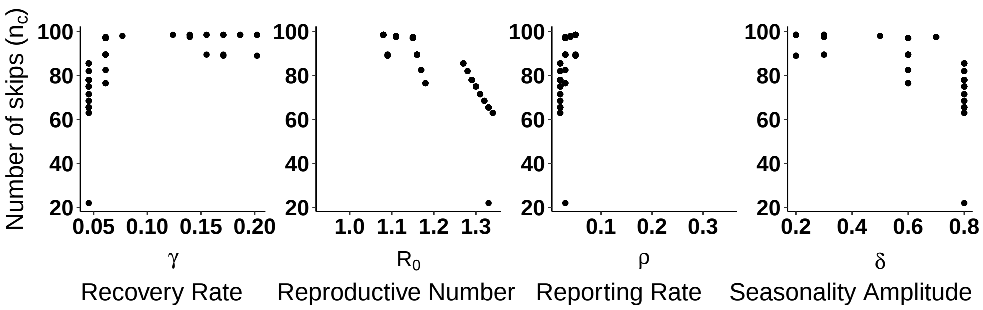

Figure 10: **Expected number of skips from deterministic calculation given all combinations of R_0, seasonal transmission amplitude (δ), and reporting rate (ρ) from each parameter combination of the recovery rate (γ) profile within 2 log likelihood units of the parameter combination with the highest likelihood in the original model.** In Figure 5 panel A, expected numbers of deterministic skips were calculated for all parameter combinations within 2 log-likelihood units from the maximum likelihood estimate of the SIR Cosine Model. Parameter values from the profile of the recovery rate (γ) in the sensitivity analysis shown in Supplemental Figure 10 were not included in the skip calculations for Figure 5 Panel A. Here, we replicate those skip calculations for all parameter combinations from the γ sensitivity analysis within 2 log-likelihood units of the original maximum likelihood estimate.

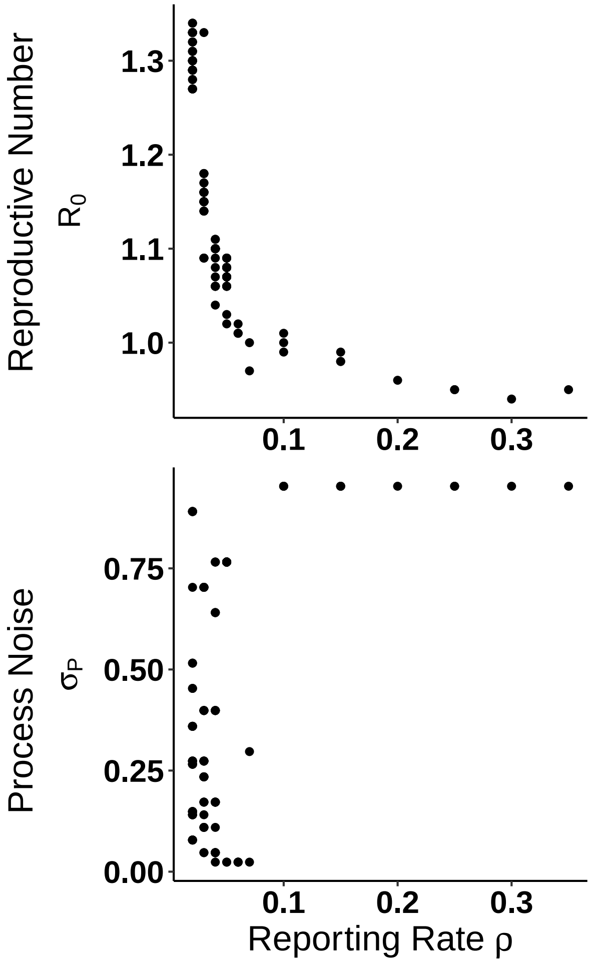

Figure 11: **Comparison of reporting rate (rho) vs. reproductive number (R_0) and environmental process noise magnitude $\sigma_{\mathbf{P}}$) for each parameter combination of the γ profile of the SIR Cosine Model within 2 log-likelihood units of the parameter combination with the highest likelihood in the original model.** This figure compares the values of R_0 and reporting rate (ρ) and process noise magntiude (σ_{P}) for all parameter combinations from the gamma profile used for the deterministic skip calculations shown in Supplemental Figure 10.

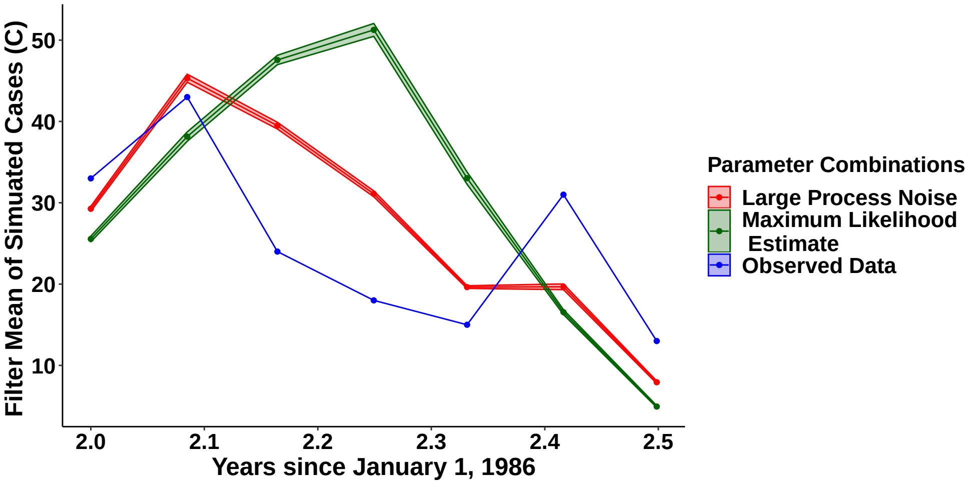

Figure 12: **Comparison of filter means between maximum likelihood parameter combination and parameter combination with large process noise over third year of simulation.** Average of filter means for the number of monthly cases (C) at each observed data point from 10 runs of the Sequential Monte Carlo algorithm pfilter run at MLE parameter combination (in green) and at the parameter combination with the highest likelihood out of all parameter combination with the highest permissible amount of process noise ($\sigma_P = 1$) shown in red. The observed cases are shown in blue for comparison. Only the third year of the fit (corresponding to the period from January 1988 through July 1988) is shown. Shaded ribbons show the average of the filter mean +/- 2 times the standard deviation of the filter means (across all 10 runs).

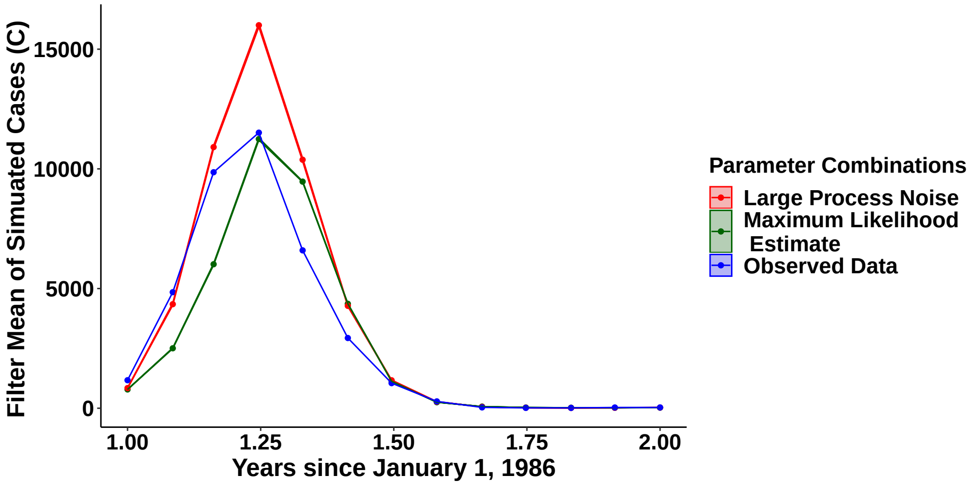

Figure 13: **Comparison of filter means between maximum likelihood parameter combination and parameter combination with large process noise over second year of simulation.** Average of filter means for the number of monthly cases (C) at each observed data point from 10 runs of the Sequential Monte Carlo algorithm pfilter run at MLE parameter combination (in green) and at the parameter combination with the highest likelihood out of all parameter combination with the highest permissible amount of process noise ($\sigma_P = 1$) shown in red. The observed cases are shown in blue for comparison. Only the second year of the fit (corresponding to the period from January 1987 through December 1987) is shown. Shaded ribbons show the average of the filter mean +/- 2 times the standard deviation of the filter means (across all 10 runs).

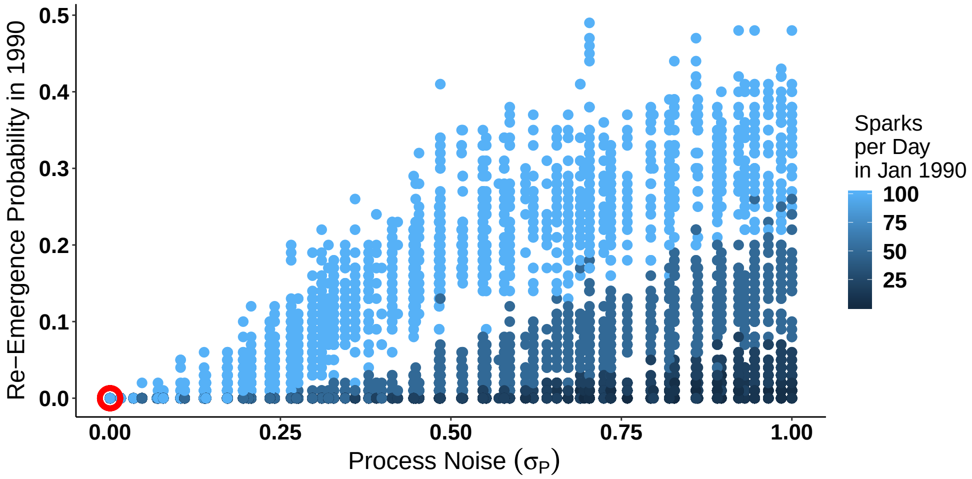

Figure 14: **Probability of stochastic epidemic in 1990 vs Process Noise Intensity (σ_P) under simulation from top 2LL parameter combinations of stochastic SIR Cosine Model.** The fitted stochastic model was simulated forward in time from 1986-1990 with population growth. Daily pulse rates of 2,5,10,20, 50, and 100 infected individuals per day in January 1990 were used. Each parameter combination within 2 log-likelihood units of the maximum likelihood estimate was simulated 100 times. The re-emergence probability was calculated by determining the number of simulations in which the susceptible population decreased in 1990. The plot shows re-emergence probability as a function of the process noise intensity σ_P. Each point represents a single parameter combination at a particular pulse rate. Points are colored by pulse rate. MLE parameter combination points are circled in red.

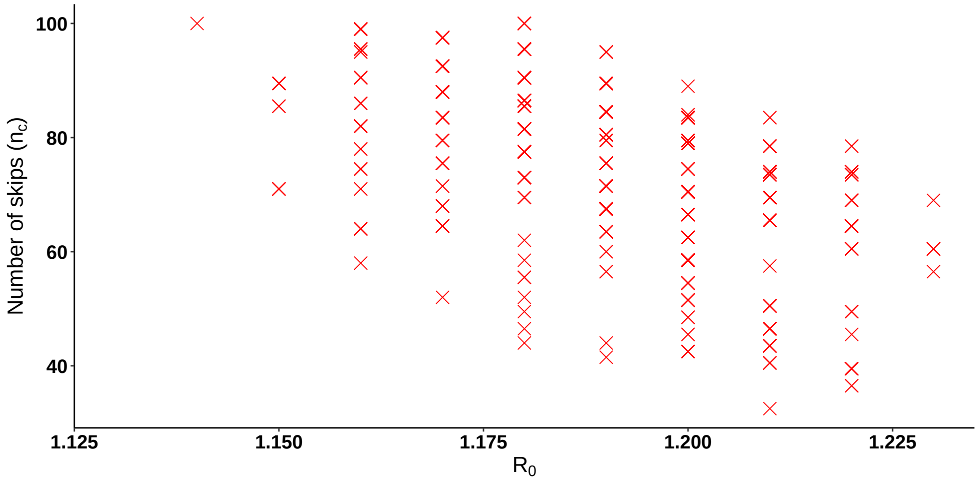

Figure 15: **Expected number of skips from deterministic calculation using parameter estimates from the fitted stochastic model.** The red crosses show the expected number of skips nc from Equation 1 of the main text using parameters and the fraction of the population susceptible after the initial DENV1 invasion (s_0) estimated from the fitted stochastic model. Each circle corresponds to one parameter combination, and we included here all parameter combinations for the fitted SIR Cosine model with different values of the reproductive number R_0, seasonal transmission amplitude δ, and reporting rate ρ. See Supplemental Figure 10 for the expected number of skips for all parameter combinations obtained from the profile of the recovery rate (γ).

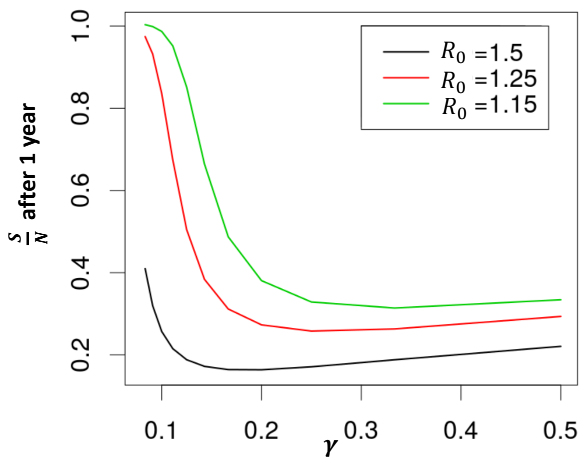

Figure 16: **Different combinations of mean transmission rate β_0 and recovery rate γ that yield the same reproductive number R_0 value have different values of the fraction of the population susceptible ($\frac{S}{N}$) after 1 year.** For example, suppose that we have the parameter combinations $\delta = 0.5$, $\beta_0 = 0.3$, $\gamma = 0.2$ and $\delta = 0.5$, $\beta_0 = 0.15$, $\gamma = 0.1$. While both parameter combinations give an R_0 of 1.5, the first yield an $\frac{S}{N}$ after one outbreak of around 20%, while the second gives an $\frac{S}{N}$ of approximately 40%. The plot shows values of $\frac{S}{N}$ as a function of γ with β_0 modified to give different values of R_0. For all points in the plot, the amplitude of seasonal transmission $\delta = 0.5$, the initial number of infected individuals $I(t = 0) = 1$, and the frequency of the seasonality of transmission $\omega = \frac{2\pi}{365}$, corresponding to an annual periodicity.

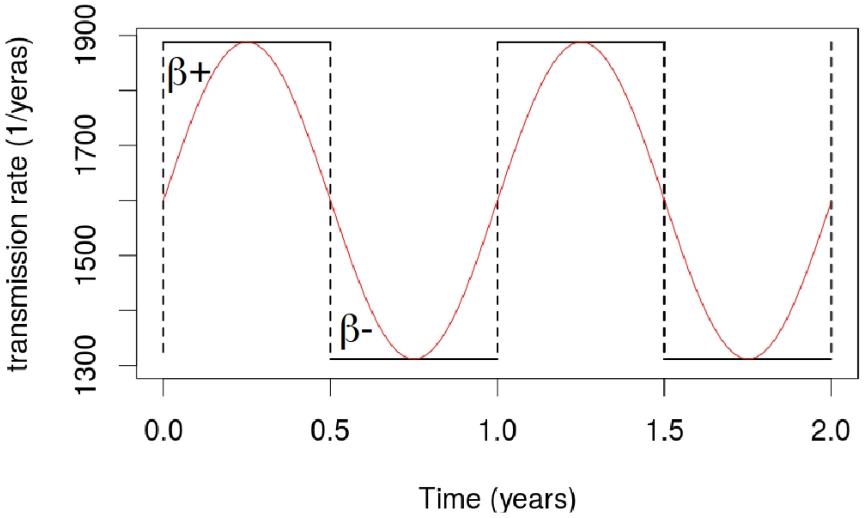

Figure 17: Transmission rate considered by Stone et al [1] in (black), and in this work (red).

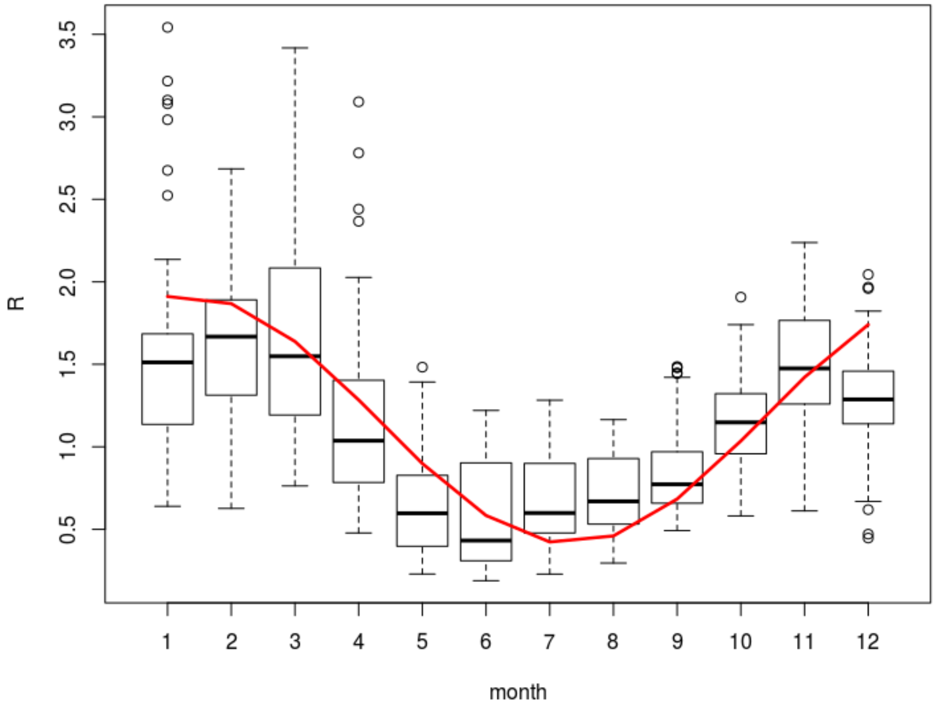

Figure 18: **A boxplot of R_0 for each month in Rio de Janeiro from 2010-2016 with monthly estimates from [13] .** The superimposed solid red line indicates the mean monthly R_0 obtained from our stochastic SIR cosine model with the parameter combination with the highest likelihood.

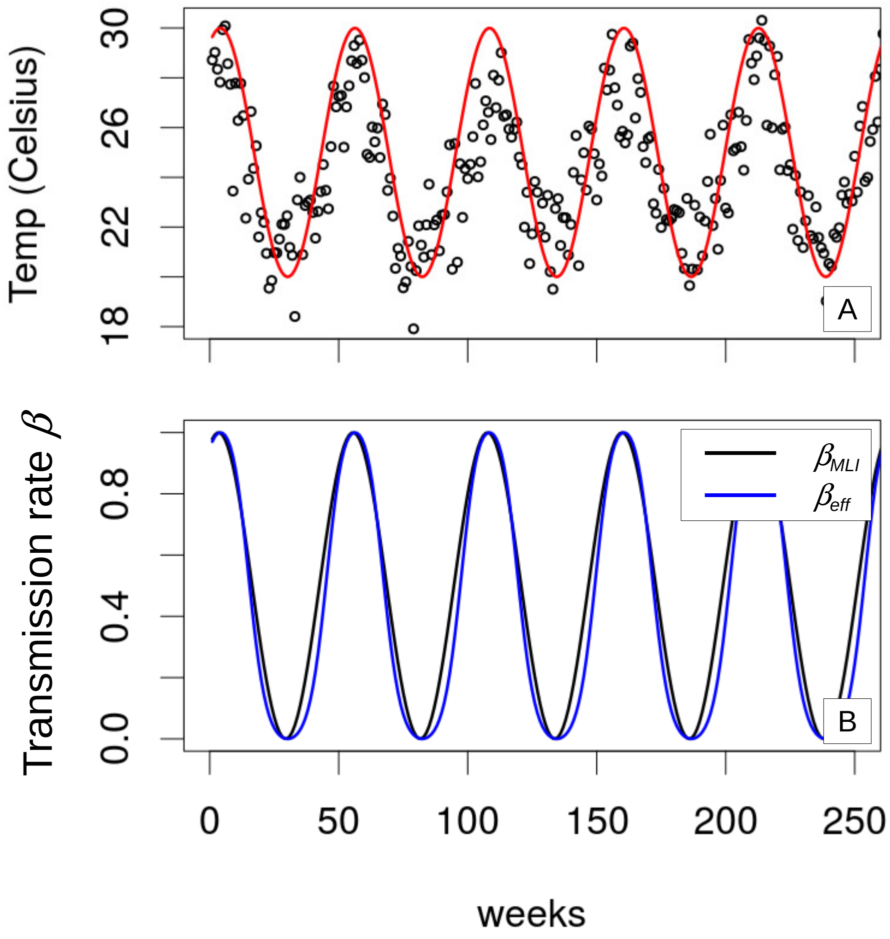

Figure 19: **A) Weekly temperature of the city of Rio de Janeiro.** The data is represented by black dots and cosine function by the solid red line. **B) Transmission rate re-scaled between 0 and 1.** The black line is the fitted sinusoidal transmission rate and the blue line the is the effective transmission rate shown on Eq.(32).

References

[1] Lewi Stone, Ronen Olinky, and Amit Huppert. Seasonal dynamics of recurrent epidemics. *Nature*, 446(7135):533–536, 2007. ISSN 1476-4687. doi: 10.1038/nature05638. URL https://doi.org/10.1038/nature05638.

[2] Brazilian Institute of Geography (IBGE) and Statistics. Brazil demographic census 2010. *Rio de Janeiro, Brazil: Brazilian Institute of Geography and Statistics (IBGE)*, 2012. URL https://censo2010.ibge.gov.br/en/noticias-censo.html?busca=1&id=1&idnoticia=2528&t=life-expectancy-at-birth-was-74-6-years-in-2012&view=noticia.

[3] Brazilian Institute of Geography (IBGE) and Statistics. "censo demographico- 1991-rio de janeiro". "Censo demogrÃąfico : 1991 : resultados do universo relativos as caracterÃŋsticas da populaÃğÃčo e dos domicÃŋlios"(Table 1.4: "PopulaÃğÃčo residente, por grupos de idade, segundo ti lolesorregiÃăes, as MicrorregiÃţes, os MunicÃŋpios,os Distritos e o sexo"):32–41, 1991. URL https://biblioteca.ibge.gov.br/biblioteca-catalogo?id=782&view=detalhes.

[4] Brazilian Institute of Geography (IBGE) and Statistics. Censo demogrÃąfico. (PopulaÃğÃčo residente, sexo e situaÃğÃčo do domicÃŋlio; Total column), 2000. URL https://ww2.ibge.gov.br/home/estatistica/populacao/censo2000/universo.php?tipo=31o/tabela13_1.shtm&paginaatual=1&uf=33&letra=R.

[5] Daihai He, Edward L Ionides, and Aaron A King. Plug-and-play inference for disease dynamics: measles in large and small populations as a case study. *Journal of the Royal Society Interface*, 7(43):271–283, 2009. ISSN 1742-5689.

[6] John H Huber, Marissa L Childs, Jamie M Caldwell, and Erin A Mordecai. Seasonal temperature variation influences climate suitability for dengue, chikungunya, and zika transmission. *PLoS neglected tropical diseases*, 12 (5):e0006451, 2018.

[7] Wolfgang Bock and Yashika Jayathunga. Optimal control and basic reproduction numbers for a compartmental spatial multipatch dengue model. *Mathematical Methods in the Applied Sciences*, 41(9):3231–3245, 2018. ISSN 0170-4214.

[8] Mathieu Andraud, Niel Hens, Christiaan Marais, and Philippe Beutels. Dynamic epidemiological models for dengue transmission: a systematic review of structural approaches. *PloS one*, 7(11), 2012.

[9] Oliver J Brady, Michael A Johansson, Carlos A Guerra, Samir Bhatt, Nick Golding, David M Pigott, HÃľlÃĺne Delatte, Marta G Grech, Paul T Leisnham, and Rafael Maciel-de Freitas. Modelling adult aedes aegypti and aedes albopictus survival at different temperatures in laboratory and field settings. *Parasites vectors*, 6(1):351, 2013. ISSN 1756-3305.

[10] Joseph PÃąez ChÃąvez, Thomas GÃűtz, Stefan Siegmund, and Karunia Putra Wijaya. An sir-dengue transmission model with seasonal effects and impulsive control. *Mathematical biosciences*, 289:29–39, 2017. ISSN 0025-5564.

[11] Tridip Sardar, Sourav Rana, and Joydev Chattopadhyay. A mathematical model of dengue transmission with memory. *Communications in Nonlinear Science and Numerical Simulation*, 22(1-3):511–525, 2015. ISSN 1007-5704.

[12] Janis Antonovics, Yoh Iwasa, and Michael P Hassell. A generalized model of parasitoid, venereal, and vector-based transmission processes. *The American Naturalist*, 145(5):661–675, 1995.

[13] Claudia T Codeço, Daniel AM Villela, and Flavio C Coelho. Estimating the effective reproduction number of dengue considering temperature-dependent generation intervals. *Epidemics*, 25:101–111, 2018.

[14] Erin A Mordecai, Jeremy M Cohen, Michelle V Evans, Prithvi Gudapati, Leah R Johnson, Catherine A Lippi, Kerri Miazgowicz, Courtney C Murdock, Jason R Rohr, Sadie J Ryan, et al. Detecting the impact of temperature on transmission of zika, dengue, and chikungunya using mechanistic models. *PLoS neglected tropical diseases*, 11(4):e0005568, 2017.

[15] Marcelo Otero, Nicolás Schweigmann, and Hernán G Solari. A stochastic spatial dynamical model for aedes aegypti. *Bulletin of mathematical biology*, 70(5):1297, 2008.
