# Supplementary material for: Predicting re-emergence times of dengue epidemics at low reproductive numbers: DENV1 in Rio de Janeiro, 1986–1990
Source: J R Soc Interface. 2020 Jun 24;17(167):20200273. doi: 10.1098/rsif.2020.0273 (PMC7328382; doi:10.1098/rsif.2020.0273)
Supplement: Supporting Information: Supplemental Tables [file rsif20200273supp2.pdf]

Predicting re-emergence times of dengue epidemics at low reproductive numbers: DENV1 in Rio de Janeiro, 1986-1990.

Supporting Information: Supplemental Tables

Rahul Subramanian, Victoria Romeo-Aznar,
Edward Ionides, Claudia T. Codeço, and Mercedes Pascual

April 2, 2020

Contents

1 Supplemental Tables **2**
1.1 Table ST1 2
1.2 Table ST2 3
1.3 Table ST3 4

1 Supplemental Tables

1.1 Table ST1

	MLE Value
sigma_P	0.00019
gamma	0.05882353
phi	1.159567
sigma_M	0.5750731
rho	0.02754872
Beta_0	0.06863794
delta	0.6458656
mu_H	3.68e-05
N_0	5281842
I_0	220312.8
R_0	0
C_0	0
r	0
omega	0.01721421
epsilon	0
LL	-165.8182
seed	338719557
Profile_Type	sigma_P
t0	120

Table 1: . MLE Parameter Values for SIR Cosine Model.

1.2 Table ST2

	SIR_Cosine	SIR_Spline	SEIR_Spline
min_Sigma_P_profile_2_LL_bound	0.00	0.00	0.00
max_Sigma_P_profile_2_LL_bound	1.00	1.00	0.69
rho_MLE	0.03	0.03	0.03
gamma_MLE	0.06	0.06	0.14
sigma_P_MLE	0.00	0.07	0.00
N_0_MLE	5281842.00	5301405.00	5301405.00
MLE_R_naught_act	1.17	1.13	1.10
min_R_naught_act_2LL	1.13	1.10	1.09
max_R_naught_act_2LL	1.24	1.16	1.14
min_sigma_P_2_LL	0.00	0.07	0.00
max_sigma_P_2_LL	1.00	0.86	0.69
min_rho_2_LL	0.02	0.02	0.02
max_rho_2_LL	0.04	0.04	0.04
min_Sigma_P_profile_2_LL_bound.1	0.00	0.00	0.00
max_Sigma_P_profile_2_LL_bound.1	1.00	1.00	0.69

Table 2: Comparison of Key Parameter Values (MLE Estimates and 2LL Profile Boundaries) for R_0, N_0, ρ, γ, and process noise parameter σ_P between several models without immigration (SIR Cosine Model , SIR Spline Model, and SEIR Spline Model). Note that γ is a fixed parameter.

1.3 Table ST3

	SEIR_Spline	SIR_Spline	SIR_Cosine
Csnippet_file_path	Csnippet_SEIR_spline_model.R	Csnippet_SIR_spline_model.R	Csnippet_SIR_cosine_model.R
Pfilter_likelihood	-165.5741	-165.5022	-165.8260
Pfilter_likelihood_se	0.01204600	0.01580957	0.01059077
AIC	347.1482	345.0043	345.6520
Num_est_parameters	8	7	7
num_years	2.5	2.5	2.5

Table 3: Likelihood Comparison and AIC scores between several models without immigration (SIR Cosine Model, SIR Spline Model, and SEIR Spline Model) with likelihoods calculated over 2 and a half years of dengue case data.
